# Heme homeostasis and its regulation by hemoproteins in bacteria

**DOI:** 10.1002/mlf2.12120

**Published:** 2024-07-11

**Authors:** Yingxi Li, Sirui Han, Haichun Gao

**Affiliations:** ^1^ Institute of Microbiology and College of Life Sciences Zhejiang University Hangzhou China

**Keywords:** heme, heme acquisition and utilization, heme export, heme homeostasis, hemoprotein

## Abstract

Heme is an important cofactor and a regulatory molecule involved in various physiological processes in virtually all living cellular organisms, and it can also serve as the primary iron source for many bacteria, particularly pathogens. However, excess heme is cytotoxic to cells. In order to meet physiological needs while preventing deleterious effects, bacteria have evolved sophisticated cellular mechanisms to maintain heme homeostasis. Recent advances in technologies have shaped our understanding of the molecular mechanisms that govern the biological processes crucial to heme homeostasis, including synthesis, acquisition, utilization, degradation, trafficking, and efflux, as well as their regulation. Central to these mechanisms is the regulation of the heme, by the heme, and for the heme. In this review, we present state‐of‐the‐art findings covering the biochemical, physiological, and structural characterization of important, newly identified hemoproteins/systems involved in heme homeostasis.

## INTRODUCTION

**Figure 1:**
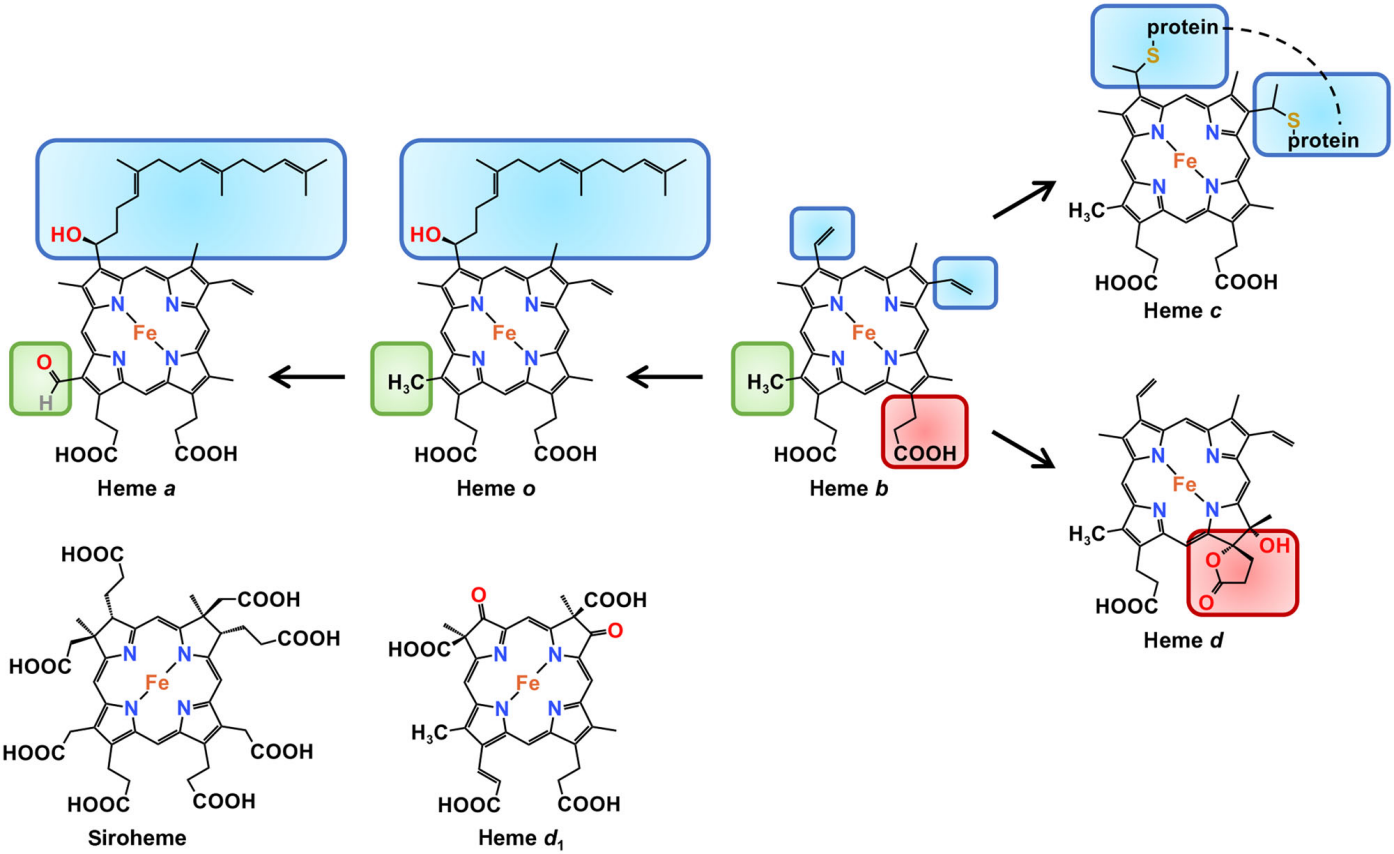
Chemical structures of hemes *a*, *b*, *c, o, d*, *d*_1_, and siroheme. Heme *b* is the prototype heme species of all known heme synthetic pathways from which other “genuine” hemes are derived, including *o*, *a*, *c*, and *d*. Differences among them are highlighted in colored boxes. Siroheme and heme *d*_1_ are not derivatives of heme *b*.

Tetrapyrroles are not only one of the most ancient prosthetic groups in all living organisms but also the most abundant pigment molecules on earth, and thus they have been dubbed “the pigments of life^1^”. A major subset of tetrapyrroles comprises many large macrocyclic compounds, among which iron‐bound protoporphyrin IX molecules are called hemes^2^. Hemes are characterized by a fully oxidized porphyrin macrocycle that contains a complete system of alternating double and single bonds, including heme *b*, heme *c*, heme *a*, and heme *o*, which vary from one to another in terms of the peripheral side chains^3^ (Figure 1). In heme‐producing organisms, the direct product of the biosynthetic pathways known to date is exclusively heme *b*, from which other heme species are derived^4^. Heme *b* is characterized by pyrrole rings A and B containing methyl and vinyl groups, whereas pyrrole rings C and D have methyl and propionate substituents^4^. While hemes *a* and *o* have a hydroxyethyl farnesyl group instead of the vinyl group on pyrrole ring A, the former additionally replaces a methyl group on ring D with a formyl group^5^, ^6^. Heme *b* can be covalently attached to certain polypeptides, and in this scenario, it gets a new name: heme *c* ^7^. Heme *d* (iron‐dihydroporphyrin) is rather atypical among the heme *b* derivates because it misses one of the double bonds on pyrrole ring C of the porphyrin and, in fact, is a member of the chlorin family^8^.

There are also some other iron‐containing cyclic modified tetrapyrroles, such as siroheme (iron‐bound isobacteriochlorin) and heme *d*_1_ (iron‐bound dioxoisobacteriochlorin), which are not considered as “genuine” hemes since they possess partially reduced pyrrole rings and are not generated through direct modification of heme *b* ^1^, ^4^ (Figure 1). For the sake of brevity and focus, this review discusses the “genuine” hemes only, and when not specified, heme refers to heme *b*.

Hemes, mostly as essential cofactors of hemoproteins, play irreplaceable roles in a myriad of physiological processes, such as oxygen transport, electron transfer, respiration, photosynthesis, oxidative stress response, signal sensing and transduction, and catalysis^9^. Hemoproteins can be divided into two distinct types, depending on the manner of heme association: type I representing those with noncovalently bound heme and type II representing those carrying one or more covalently attached heme(s)^7^. Most type II hemoproteins are called cytochrome *c* (cyt *c*), and the heme attachment process, namely, cyt *c* maturation (Ccm) or biosynthesis, is a posttranslational modification catalyzed by one of three specific enzymatic systems, depending on bacterial species^10^. For each heme within cyt *c*, there are two thioether bonds between the cysteine thiols of a heme‐binding motif (HBM), typically CXXCH, where X represents any residue, and the vinyl side chains of heme^10^. A few proteins known to have heme attached via covalent bonds to other amino acid residues include CcmE (histidine), which is a component of the Ccm system (System I) acting as a heme chaperone, *Synechocystis* hemoglobin (Hb) (histidine), and cyt P460 of *Nitrosomonas europea* (lysine in addition to two cysteine residues)^11^, ^12^, ^13^. In addition to sequestered heme within hemoproteins, exchangeable heme (also called labile heme, which is called heme if not otherwise noted in this review for simplicity) serves as an accessible supply of heme to target proteins in the cell^14^. Paradoxically, heme has the potential to cause cytotoxicity at high concentrations^15^. In eukaryotes, excess heme is able to readily interact with lipophilic membranes and enter membrane‐enclosed compartments, triggering radical chain reactions to generate reactive oxygen species (ROS), oxidative injury, inflammation, and immune dysfunction^16^, ^17^. Similarly, when excess heme accumulates in bacterial cells, it may also promote ROS generation, leading to membrane peroxidation and damage to cellular proteins and nucleic acids^15^, ^18^, ^19^, ^20^. This is particularly true for many pathogens that may be exposed to high concentrations of heme from lysed erythrocytes^18^. Therefore, tight control of the intracellular pool of heme is required for the balanced regulation of heme in the cell, that is, heme homeostasis.

Heme homeostasis within cells is maintained primarily by balancing the rates of heme biosynthesis/uptake and catabolism/export^14^. Although all these processes have been under investigation for decades, many significant discoveries have been made fairly recently. In this review, to expand our understanding of heme homeostasis in bacteria, we will highlight the latest advances in most processes critically associated with heme, including biosynthesis, acquisition, utilization, intracellular trafficking, export, and degradation, as well as their regulation by hemoproteins.

## BIOSYNTHESIS OF HEME

**Figure 2:**
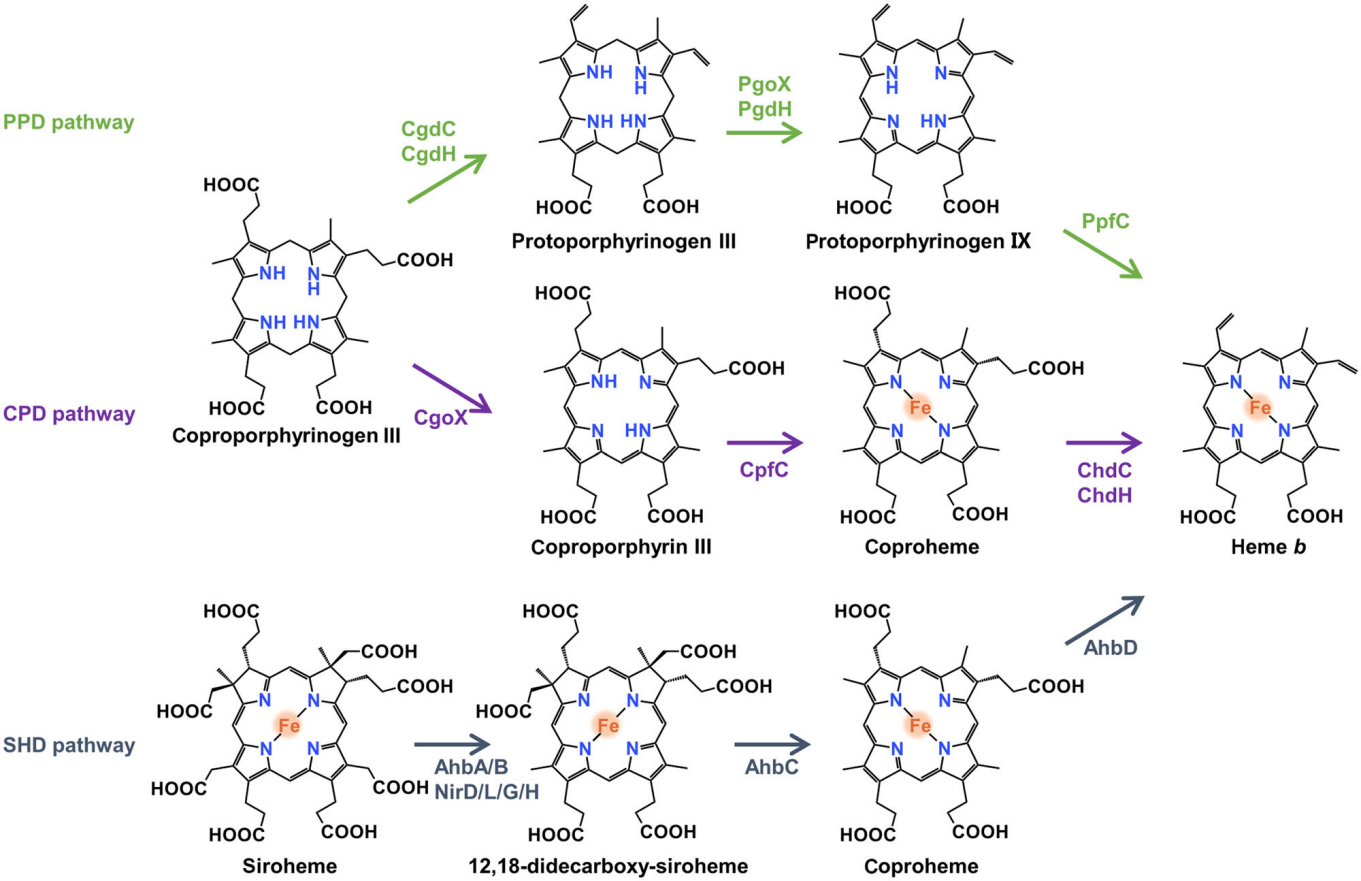
Heme biosynthetic pathways found in bacteria. The steps that are conserved in both eukaryotes and prokaryotes leading to the production of the common intermediate uroporphyrinogen III are not shown. From uroporphyrinogen III, heme biosynthesis in bacteria can be carried out through three distinct pathways: protoporphyrin‐dependent (PPD), coproporphyrin‐dependent (CPD), and siroheme‐dependent (SHD) pathway.

In recent years, how prokaryotes synthesize hemes has been summarized and reviewed elegantly and comprehensively^2^, ^3^, ^4^, ^21^. Therefore, we will discuss this subject only briefly. While it is clear that all eukaryotes capable of synthesizing heme endogenously utilize the same route, at least three pathways have been identified in prokaryotes^22^ (Figure 2). Despite this, 5‐aminolevulinic acid (5‐ALA) serves as the universal precursor metabolite^4^. There are two ways to generate 5‐ALA: the C4 and C5 pathways. While the C4 pathway is present in some α‐proteobacteria and most eukaryotic organism but not in higher plants, the C5 pathway occurs in most prokaryotes and higher plants^2^. The C4 pathway entails a single enzyme, 5‐ALA synthase (AlaS), which utilizes pyridoxal phosphate (PLP) as a cofactor to catalyze the condensation of succinyl‐CoA and glycine to form 5‐ALA^23^, ^24^. In contrast, the C5‐pathway uses two enzymes for 5‐ALA formation, with glutamyl‐tRNA as the initial substrate^25^. Glutamyl‐tRNA reductase (GtrR) catalyzes the transformation of glutamyl‐tRNA into glutamate‐1‐semialdehyde (GSA). Subsequently, GSA aminomutase (GsaM) converts GSA into 5‐ALA using pyridoxamine‐5′‐phosphate as a cofactor. Interestingly, although the C4 pathway is simpler, it is not superior to the C5 pathway for heme production^26^. It should be mentioned that 5‐ALA serves as the key substrate that largely dictates overall heme synthesis in vivo^27^. Conceivably, AlaS is generally subjected to feedback inhibition by heme^28^.

The formation of uroporphyrinogen III from 5‐ALA relies on three reactions catalyzed by three enzymes, which are perfectly conserved in all organisms that are able to synthesize heme endogenously^2^. After uroporphyrinogen III, prokaryotic organisms can synthesize hemes via one of three distinct pathways (Figure 2). The first is called the protoporphyrin‐dependent (PPD) pathway, which is mostly present in Gram‐negative bacteria and is the only route operating in eukaryotes^4^. In contrast, many Gram‐positive bacteria are mainly equipped with the coproporphyrin‐dependent (CPD) pathway. These two pathways share the first step, the formation of coproporphyrinogen III from uroporphyrinogen III, but they differ from each other in the subsequent three steps^29^. The last one is found in some denitrifying and sulfate‐reducing bacteria and archaea, namely, the siroheme‐dependent (SHD) pathway, involving the conversion of uroporphyrinogen III to siroheme, from which the synthesis of heme and heme *d*_1_ diverges^30^ (Figure 2).

## ACQUISITION OF HEME

Given that heme is an iron‐containing molecule essential for various biological functions, bacteria have evolved the ability to acquire heme from their surroundings for survival and fitness. This capacity is particularly crucial for heme auxotrophs, which were previously believed to be limited to pathogens, symbionts, or microorganisms living under nutrient‐replete conditions but have been recently found to be widespread in ubiquitous, free‐living, aquatic bacterial groups^31^, ^32^, ^33^. To date, a variety of heme acquisition strategies have been identified and characterized in bacteria. It should be noted that Gram‐negative and ‐positive bacteria can use drastically different systems for heme uptake because of the presence of the outer membrane (OM) in Gram‐negative bacteria, which represents an additional barrier to heme acquisition.

**Figure 3:**
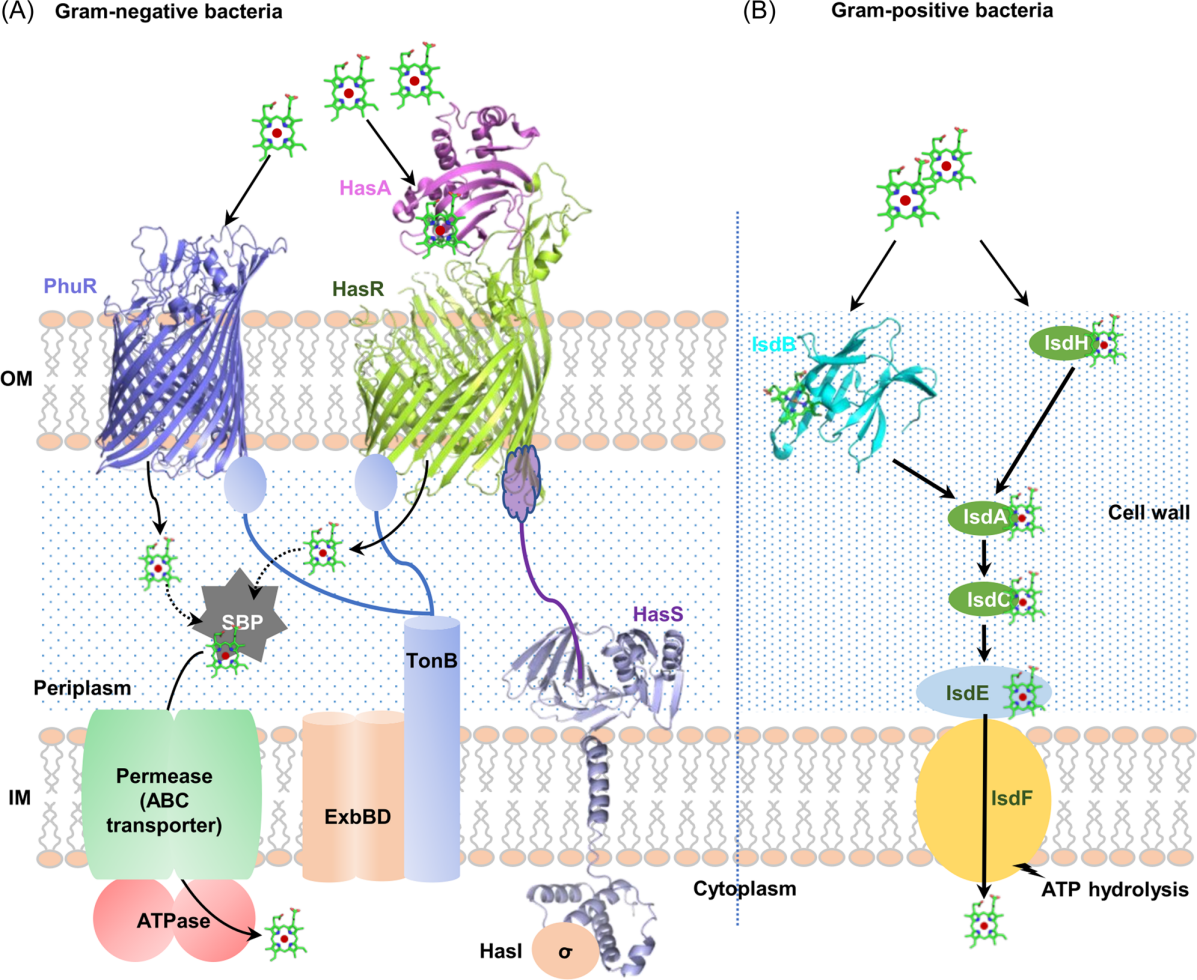
Schematic overview of heme acquisition in Gram‐negative bacteria and Gram‐positive bacteria. (A) Representative heme acquisition systems in Gram‐negative bacteria. The heme is shown as a green ball‐stick model. Heme translocation pathways across the outer membrane (OM) mediated by type I and type II TonB‐dependent receptors (TBDRs), represented by *Pseudomonas aeruginosa* PhuR and *Serratia marcescens* HasR (together with hemophore HasA), respectively (PDB ID: 5C58), are shown. The ABC transporter, composed of periplasmic SBP (a substrate‐binding protein), inner membrane (IM)‐bound permease, and cytoplasmic ATPase, is responsible for heme translocation across the IM. HasA/HasR also functions as a heme sensor for the HasS/HasI regulatory system. Upon activation, anti‐σ HasS releases σ HasI, allowing the latter to direct transcription of its target genes. (B) Iron‐regulated surface determinant (Isd) system for Gram‐positive bacteria heme acquisition in *Staphylococcus aureus* (IsdB, PDB ID: 3RUR).

In Gram‐negative bacteria, the most widespread heme uptake strategy involves a TonB‐dependent receptor (TBDR) and an ATP‐binding cassette (ABC) transporter for translocating heme across the OM and the inner membrane (IM), respectively^34^ (Figure 3A). TBDRs are ubiquitous OM β‐barrel proteins that recognize and transport their substrates with high affinity, spending energy derived from the proton motive force transmitted from the TonB−ExbB−ExbD complex located in the IM^35^. There are two types of bacterial TBDRs specific to heme uptake, and they frequently coexist. While type I TBDRs capture hemes directly from the extracellular milieu, type II requires accessory proteins, such as hemophores and surface‐anchored lipoproteins (SLPs)^36^.

Type I TBDRs have a highly conserved overall architecture, composed of 22 antiparallel β‐strands and an N‐terminal plug domain, such as HasR and PhuR of *Pseudomonas aeruginosa*, Hma, ChuA of uropathogenic *Escherichia coli*, and PutA of *Shewanella oneidensis*, to name a few^37^, ^38^, ^39^, ^40^, ^41^. To ensure heme recognition and binding with high accuracy, these TBDRs commonly utilize histidine and tyrosine residues to coordinate the heme with a millimolar *K*_d_ (e.g., Hma, *K*_d_ = 8 × 10^−6^ M)^38^, ^42^ (Figure 3A).

Most type II TBDRs characterized thus far interact with hemophores, a group of proteins that are secreted by bacterial cells to scavenge free heme or extract heme from type I hemoproteins, such as hemoglobin, with a subnanomolar *K*_d_. HasA‐like hemophores, named after the structure‐available 19 kDa HasA of *Serratia marcescens* (*K*_d_ = 1.8 × 10^−11^ M), are widely distributed and conserved^43^, ^44^, ^45^ (Figure 3A). In *S. marcescens* and *P. aeruginosa*, HasA proteins bind heme and deliver it to the cognate TBDR HasR, which works with TonB−ExbB−ExbD to internalize the heme, the same as type I TBDRs^46^, ^47^, ^48^, ^49^. In addition, HasA and HasR are also essential components of a regulatory cascade that controls the transcription of many genes involved in heme homeostasis; this will be considered later. Apart from HasA, hemophores found in Gram‐negative and ‐positive bacteria belong to several protein families that lack sequence similarity^50^. For example, HxuA‐like hemophores, approximately 100 kDa and restricted to the *Pasteurellaceae* genus, are anchored at the surface of the OM to capture and deliver heme‐hemopexin (a serum glycoprotein with very high heme affinity) to cognate TBDRs^51^. HasA‐ and HxuA‐like hemophores differ, in that the latter promote heme release from hemopexin without having a detectable affinity for heme^52^. Additionally, some hemophores are OM‐bound, which can be released from the membrane by specific proteases, such as *Porphyromonas gingivalis* HmuY, presumably as a means of increasing efficiency in binding and delivering heme to their cognate TBDRs^53^, ^54^. Intriguingly, hemophores can be secreted across the OM by different secretion pathways: for example, HasA of *S. marcescens* by a type I secretion pathway, HxuA of *Haemophilus influenzae* by its specific TpsB partner, and HphA of *Acinetobacter baumannii* by a Slam protein^55^, ^56^, ^57^.

Once in the periplasm, the heme is rapidly captured by a substrate‐binding protein (SBP), usually the subunit of the ABC transporter system that transports the heme across the IM into the cytoplasm^58^. These proteins bind heme with high affinity, only modestly inferior to hemophores^59^. Many heme SBPs have been studied in terms of biochemistry and structure, such as HemT of HemTUV in *S. marcescens*, PhuT (*K*_d_ = 1.2 × 10^−9^ M) of PhuTUV in *P. aeruginosa*, ChuT of ChuTUV in uropathogenic *E. coli*, HutB of HutBCD in *Vibrio cholerae*, HmuT (*K*_d_ = 2.9 × 10^−10^ M) of HmuTUV in *Yersinia pestis*, and BhuT of BhuTUV of *Burkholderia cenocepacia* ^60^, ^61^, ^62^, ^63^. After capturing the heme, holo‐SBP interacts with the permease of the ABC transporter, leading to the activation of the ATPase component, which, in turn, induces large‐scale conformational changes in the permease to facilitate the uptake of heme^18^. Despite the presence of a common minimum architecture among these ABC transporters, the molecular mechanisms of ATP binding and hydrolysis that mediate heme translocation across the IM are remarkably diverse^64^. In addition, there are promiscuous ABC transporters that can import hemes from the periplasm to the cytoplasm. ABC transport systems for short peptides are well recognized, including Dpp (dipeptide) in *E. coli* and *Mycobacterium tuberculosis*, Opp (oligopeptide), and Sap (sensitivity to antimicrobial peptides) in *H. influenzae* ^65^, ^66^, ^67^, ^68^.

Gram‐positive bacteria lack OM and have evolved cell wall‐anchored heme‐binding receptors that are utilized for heme uptake instead of TBDRs^20^. In *Staphylococcus aureus*, heme acquisition is mediated by the iron‐regulated surface determinant (Isd) system, which is considered a heme‐uptake prototype for Firmicutes^20^ (Figure 3B). During infection, erythrocytes are lysed to release Hb into the bloodstream, producing accessible heme, including free heme, Hb, and the complex of Hb–Hp (haptoglobin)^69^. Multiple receptors, IsdA, IsdB, IsdC, and IsdH, which resemble hemophores or SBPs that function in Gram‐negative bacteria, carry out heme capture through conserved near transporter (NEAT) domains and heme transfer in the order of IsdB/IsdH, IsdA, and IsdC^70^, ^71^, ^72^. The NEAT domain is constituted by a conserved eight‐stranded β‐sandwich fold and is responsible for binding heme, Hb, and Hb‐Hp^71^, ^72^. The numbers of NEAT domains in these Isd receptors vary, presumably explaining their different binding affinities^73^. IsdC delivers heme to the heme‐specific lipoprotein IsdE, which interacts with the permease IsdF to transfer heme across the membrane, a process depending on the energy released from ATP hydrolysis catalyzed by housekeeping but nonspecific ATPase FhuC^74^. In contrast, *Corynebacterium diphtheriae*, a pathogen that causes diphtheria, has a heme uptake system composed of a dedicated ABC transporter HmuTUV (HmuT, the heme‐binding protein; HmuU, the permease; HmuV, the ATPase) and a couple of cell wall‐anchored heme‐binding receptors, HtaA and HtaB^75^, ^76^, ^77^. HtaA acquires heme from Hb and various other hemoproteins, and then transfers it to HtaB. Both HtaA and HtaB contain conserved region (CR) domains that bind hemes, which have no similarity to the NEAT domain^78^, ^79^. Recently, additional heme‐binding proteins have been identified in *C. diphtheriae*, including the ChtA/C proteins that share sequence similarity with HtaA in the CR domains, and HbpA, which lacks the CR domain but can uptake heme from Hb–Hp^80^. Thus, it appears that heme uptake by Gram‐positive bacteria is carried out through diverse pathways and involves multiple surface proteins.

## UTILIZATION AND DEGRADATION OF HEME

**Figure 4:**
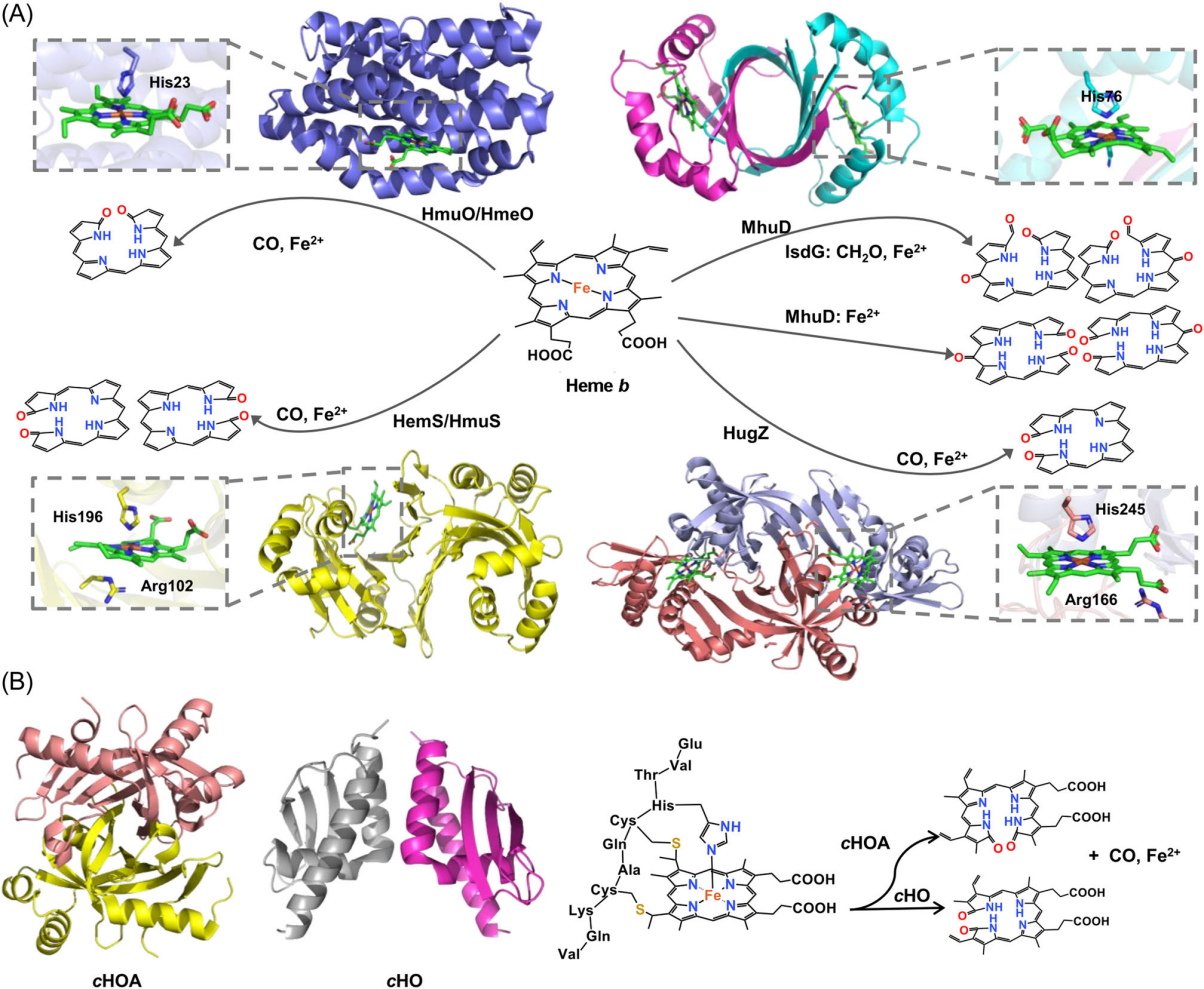
Heme degradation in bacteria. (A) Representative heme oxygenases from four distinct groups: *Corynebacterium diphtheriae* HmuO (PDB ID: 1IW0), *Mycobacterium tuberculosis* MhuD (PDB ID: 4NL5), *Yersinia enterocolitica* HemS (PDB ID: 2J0P), and *Helicobacter pylori* HugZ (PDB ID: 3GAS). The resultant products of heme degradation, biliverdin, mycobilin, or staphylobilin are shown in the chemical structures. (B) Heme *c* oxygenases. *c*HOA (PDB ID: 6VNA) and *c*HO were predicted using Colabfold. The heme is shown as a green ball‐stick model. The resultant products of heme degradation are shown in the chemical structures. *c*HOA, *c*‐type heme oxygenase A.

To utilize iron from the heme imported from the surroundings, diverse heme oxygenases (HOs) are known in bacteria to release the tightly bound iron from the heme, varying from one to another in the structural folds and in the regiospecificity of the ring cleavage reactions^81^. Not surprisingly, the genes for HOs are often clustered with those for heme uptake (Figure 4A,B). In fact, HOs play a more profound role in maintaining intracellular heme homeostasis because they also degrade heme synthesized endogenously, thereby affecting physiological processes required for survival and growth under normal conditions^82^, ^83^.

HOs can be classified into four groups based on sequence similarity and structural characteristics^82^, ^83^ (Figure 4A,B). The first HO group includes eukaryotic HO‐1, which was characterized nearly six decades ago, and its prokaryotic homologs, such as those from *C. diphtheriae* (HmuO) and *Neisseria meningitidis* (HemO)^82^, ^84^ (Figure 4A). HOs in this group break heme at the α‐meso position, resulting in biliverdin IXα, ferrous iron, and carbon monoxide (CO)^75^, ^85^. HOs belonging to the second group are found exclusively in Gram‐positive bacteria, for example, *S. aureus* IsdG and *M. tuberculosis* MhuD^86^, ^87^. While these enzymes may cleave heme at different positions to open the ring, the resultant products have an oxo group at the unopened β‐ or δ‐meso position in common^81^. The HOs of the third group studied are mostly from the *Yersinia* genus (HemS or HmuS), which cleaves the heme at β‐ or δ‐meso positions, producing a mixture of β‐ and δ‐biliverdins^88^, ^89^. The last group of HOs known to date is widely distributed, but only the HugZ of *Helicobacter pylori* has been characterized biochemically and structurally^90^. Although HugZ and HmuS differ substantially from each other in structure, they cleave the heme at the same positions and generate the same products^89^. It should be noted that there are always exceptions. For example, PigA of *P. aeruginosa*, a member of the first group, cuts heme at β‐ or δ‐meso positions, whereas *E. coli* ChuS, which belongs to the third group, breaks them at two adjacent meso positions simultaneously, resulting in a tripyrrole and a hematinic acid^91^, ^92^.

Most recently, a couple of HOs have been unveiled to specifically degrade heme *c*, including HugZ homolog *c*HOA (*c*‐type heme oxygenase A) from *Paracoccus denitrificans* and *c*HO from *Synechocystis* sp. 6803^83^, ^93^. *c*HOA catalyzes the oxidative cleavage of heme *c* at β‐ or δ‐meso positions that are covalently attached to a short peptide, which comes from the protease digestion of a natural cyt *c*. The resulting products, similar to those of HugZ, include peptide‐linked biliverdin, CO, and ferrous iron^83^ (Figure 4B). In contrast, *c*HO breaks the heme at the α‐meso position and catalyzes the cleavage of the thioether bond, releasing biliverdin IXα, peptide, ferrous iron, and CO^93^ (Figure 4B). In both cases, no heme‐degrading activity toward heme *b* was observed. HugZ and *c*HOA appear structurally similar, but the latter loses the C‐terminal loop and the enlarged opening of the active site. This difference has been proposed to account for the change in substrate specificity from heme *b* to heme *c* on a short peptide^83^. In *P. denitrificans*, the *choA* gene is the last gene of a six‐gene operon (Opp), the first five of which are primarily responsible for oligopeptide utilization. Given that such peptide transport systems have been established to play a role in heme uptake, it is logical to speculate that oligopeptides with heme *c* are imported into the cytoplasm from the periplasm by the Opp system, and *c*HOA functions to release iron^65^, ^68^, ^83^. In addition, the HupZ of Group A *streptococcus* (GAS) has been demonstrated to have heme *c* oxygenase activity, albeit not specifically^94^. Moreover, HOs sharing similarities in structure but not in sequence with *c*HOA are widely distributed, such as the HutZ of *V. cholerae*. Given that HutZ shows a rather weak activity of heme *b* degradation, producing typical products with regiospecificity similar to HugZ, it has been proposed that HutZ and HupZ may also prefer to degrade heme *c* ^83^, ^95^.

The discovery of heme *c* oxygenases is significant, especially for prokaryotes, because they generally produce many diverse cyts *c*, type II hemoproteins^10^. Some bacteria are well known for respiratory versatility, a feature largely attributed to cyts *c*, for example, 111 and 41 for *Geobactor sulfurreducens* and *S. oneidensis*, respectively, two research models for extracellular electron transport and respiration^96^. Cyts *c*, which are exclusively membrane‐bound or soluble in the periplasm to function as electron carriers and/or oxidoreductive enzymes in general, are synthesized in response to environmental cues, such as the availability of certain electron donors and acceptors^97^, ^98^. Thus, in bacteria expressing a large repertoire of cyts *c*, the turnover of these proteins with associated hemes may be critical for in vivo fitness, especially when iron is limited.

## INTRACELLULAR HEME TRAFFICKING

Eukaryotic cells comprise intracellular organelles and are therefore compartmentalized. As heme biosynthesis occurs in mitochondria, but most hemoproteins function elsewhere, it is conceivable that eukaryotic cells must mobilize heme from the site of synthesis to heme‐dependent proteins in the cytosol, associated with other organelles or not, and the nucleus. A common view is that heme chaperones are involved in allocating exchangeable hemes to eukaryotic cells^99^, ^100^, ^101^, ^102^, ^103^, ^104^. In contrast, whether heme trafficking occurs in bacteria is not self‐evident, given that bacteria generally lack intracellular organelles, and heme is synthesized in the same compartment where most type I hemoproteins reside and function.

To date, only a few cytoplasmic bacterial proteins, HemW (*K*_d_ = 2 × 10^−5^ M) from *E. coli*, Cj1386 (*K*_d_ = 6.99 × 10^−9^ M) and CgdH2 from *Campylobacter jejuni*, and HtpA (*K*_d_ = 1.2 × 10^−6^ M) from *S. oneidensis*, have been demonstrated to be able to traffic heme intracellularly^105^, ^106^. HemW is modestly homologous (33% amino acid sequence identity) to bacterial HemN, coproporphyrinogen III dehydrogenase, both of which belong to the radical SAM superfamily. Compared to HemN, however, HemW is truncated in the N terminus, lacks the fourth cysteine of the conserved CX_3_CX_2_CXC motif, which is the signature of all HemW‐like proteins^105^, and does not have radical SAM enzyme activity. Instead, HemW is able to bind heme with a stoichiometry of 1:1 and delivers it to hemoproteins bacterioferritin and NarI, a subunit of nitrate reductase NarGHI^106^. Not surprisingly, the human HemW homolog RSAD1 is also a heme‐binding protein^106^.

Cj1386 from *C. jejuni* binds to heme, and one of its downstream targets is hemoprotein catalase KatA^107^, ^108^. Cj1386 shows the hexacoordinated heme‐binding ability by Tyr57 with a 1:1 ratio, which confirms that the Y57A mutation results in reduced activity and heme content in KatA. *C. jejuni* also encodes another heme‐trafficking protein, CgdH2, which is not homologous to Cj1386^109^. CgdH2 binds heme by residues His45 and His133, with a dissociation constant of 4.9 ± 1.0 μM, and facilitates heme transfer to ferrochelatase^107^. CgdH2 also belongs to the radical SAM superfamily but is evolutionarily distinct from currently known chaperones, such as HemW^110^.

**Figure 5:**
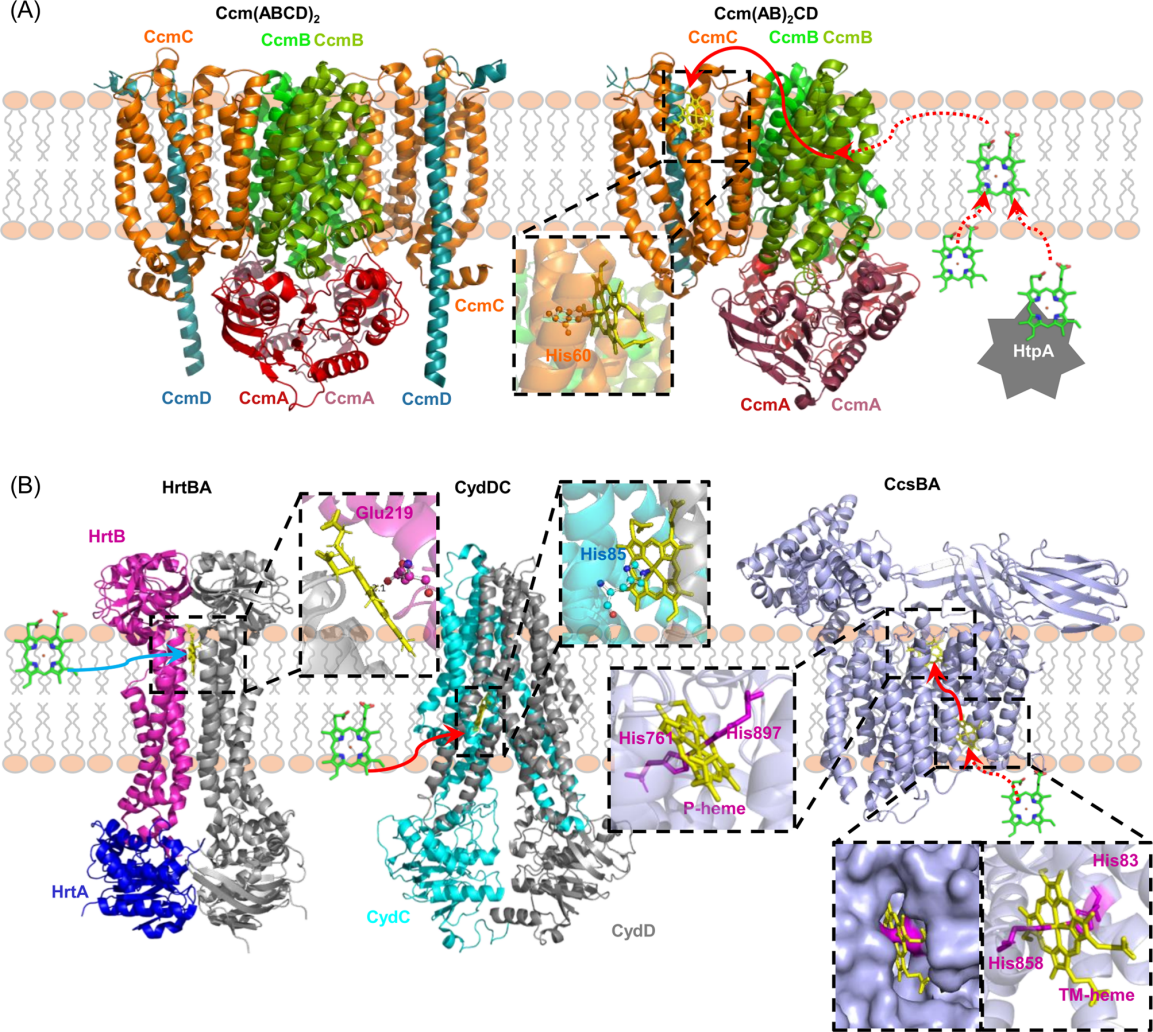
Heme exports in bacteria. The heme in the cytoplasm and the membrane is shown as a green ball‐stick model, while the transporter is shown as a yellow ball‐stick model. The transport routes of heme substantiated experimentally and proposed are shown by solid and dashed arrow lines, respectively. (A) Structures of HrtBA, CydDC, and CcsBA. *Corynebacterium diphtheriae* HrtBA is heterodimer (PDB ID: 7W7D), with one monomer in gray and the other in magenta and blue; *Escherichia coli* CydDC is heterodimer (PDB ID: 8IPS), with CydC in cyan and CydD in gray; *Helicobacter hepaticus* CcsBA is in open conformation (PDB ID: 7S9Y). Heme could be present at two sites. The surface image shows a side view of heme binding. (B) The CcmABCD system in two cryo‐EM structures (PDB ID: 8CE1, 7F04) with different complex compositions, Ccm(ABCD)_2_ or Ccm(AB)_2_CD. The CcmA subunits (red and raspberry), CcmB subunits (green and splitpea), CcmC (orange), and CcmD (deep teal) are shown. After synthesis, hemes may enter the membrane autonomously or with the help of the heme‐trafficking protein HtpA. CcmABCD translocates heme to CcmC from the cytoplasm, or likelier from the membrane.

HtpA proteins are widely distributed across all domains of cellular organisms, and even a few viruses. In eukaryotes, they are called TANGO2 (transport *an*d Golgi organization), which was initially identified in *Drosophila melanogaster* ^111^. HtpA and TANGO2 proteins from multiple organisms are able to bind heme with a 1:1 stoichiometry and affinities similar to those of heme‐trafficking proteins (*K*_d_ = 1.5 × 10^−5^ M for yeast TANGO2)^112^, ^113^. More importantly, these proteins can deliver heme to multiple downstream hemoproteins, such as catalase, and the Ccm system^112^, ^113^ (Figure 5A). However, although HtpA regulates the activity of catalase KatB, the direct interaction between them remains elusive, which is also true for HemW^106^, ^114^, ^115^. Therefore, heme trafficking proteins in bacteria, such as HemW and HtpA/TANGO2, likely cause weak and often highly transient interactions with their protein targets. Intriguingly, most bacterial homologs are unable to functionally replace HtpA in *S. oneidensis*, let alone TANGO2 proteins, presumably due to parallel molecular evolution. It seems that a common ancestor possessing the heme‐binding trait gives rise to all modern HtpA/TANGO2 proteins, which have evolved mechanistic differences that are sufficiently significant to amount to nonexchangeability in functionality^113^.

## EXPORT OF HEME IN BACTERIA

Heme export has been regarded as an important branch of heme homeostasis^20^. Although the most common strategy to alleviate the cytotoxicity of excess heme is degradation, bacteria are generally equipped with systems that are capable of exporting heme out of the cell directly^116^. To date, two types of heme export systems have been identified in bacteria: the ABC transporter and the major‐facilitator superfamily (MFS) transporter^117^. While only one MFS transporter is reported and mechanistic insights into how it works are lacking^118^, multiple ABC transporter heme efflux systems have been extensively studied and will be discussed here.

The best characterized heme export system that confers bacterial cell heme resistance is HrtBA (heme‐regulated ABC transporter, with transmembrane HrtB and cytoplasmic HrtA as permease and ATPase, respectively), encoded in many Gram‐positive bacteria, pathogens in particular, such as *S. aureus*, *C. diphtheriae*, and *Enterococcus faecalis*, to name a few^119^, ^120^, ^121^, ^122^ (Figure 5B). Due to the lack of OM barriers, the cytoplasmic membranes of Gram‐positive bacteria are often exposed to high concentrations of heme released from lysed erythrocytes. As heme is hydrophobic and lipophilic, it can easily accumulate in the membrane, rendering the membrane a primary target of heme toxicity^123^. To confer resistance to the heme, HrtBA functions as a heme efflux pump, detoxifying the heme by expelling it from the membrane rather than transporting it from the cytoplasm to the extracellular space. Each HrtBA complex consists of two HrtA and two HrtB subunits, and a heme‐binding pocket is formed in the HrtB dimer^124^. When extracting the heme from the membrane, Glu219 of either HrtB subunit is used as an axial ligand to keep the heme within the transmembrane helix bundle. Subsequently, the binding of ATP to HrtA induces a conformational change in HrtBA, which leads to expelling the heme from the pocket. In the end, ATP hydrolysis resets HrtBA to the unliganded state for the next round of heme export^124^, ^125^ (Figure 5A).

The homologs of HrtBA have yet to be identified in Gram‐negative bacteria. This may seem reasonable, given that the OM is not permeable to heme. By the same token, whether Gram‐negative bacteria have evolved heme efflux pumps is an interesting question to answer in the future. However, Gram‐negative bacteria must possess heme‐transporting systems that are able to translocate the newly synthesized heme from the cytoplasm to the periplasm, where heme‐dependent protein complexes assemble. CydDC, whose exact function has been under debate for decades, has recently been substantiated to be a heme ABC transporter^126^, ^127^. CydDC structures by cryogenic electron microscopy (Cryo‐EM) demonstrate that CydDC captures heme from the IM and releases it to the periplasm for the assembly of cyt *bd* oxidase^127^ (Figure 5B). In most bacteria, cyt *bd* oxidase is composed of three transmembrane subunits (CydABX) and functions as an auxiliary oxygen reductase (traditionally called terminal oxidase) found exclusively in bacteria, conferring cell resistance to a variety of harmful chemicals^128^, ^129^, ^130^. Heme initially binds within a cavity formed by transmembrane helices of CydDC, which is in the vicinity of the lateral IM plane, and primarily interacts with the invariant His85 of CydC, functioning as an axial ligand (Figure 5B). After ATP binding, a conformational change takes place, allowing CydDC to translocate heme from IM to cyt *bd* oxidase^127^.

Another example is the Ccm system, which catalyzes the covalent attachment of heme to the polypeptide chain of apo‐cyt *c* ^10^. Three distinct Ccm systems are found in eukaryotes and prokaryotes: System I (CcmABCDEFGH(I)) (Figure 5A), System II (CcsBA), and System III (HCCS). The CcsBA of System II, a cyt *c* synthetase that also functions in heme transport^131^, has recently been shown to translocate heme from the cytoplasmic side of the IM to the periplasmic side^132^ (Figure 5B). In CcsBA, a heme‐binding site formed by transmembrane helices is present near the inner leaflet of the IM, presumably taking heme from the cytoplasm^132^. Importantly, there is a clear path that could allow heme to travel from this heme site to the external heme site, which is close to the periplasm, and the highly conserved tryptophan‐rich loop, called the WWD domain, which holds heme^131^. Intriguingly, heme translocation in CcsBA appears to be an autonomous process because CcsBA lacks an ATPase domain^133^.

System I complexes, which are widely distributed in diverse Gram‐negative bacteria and archaea, as well as in plant and protozoan mitochondria, consist of eight or nine proteins organized into two functional modules: CcmABCDE and CcmFGH(I)^10^, ^134^, ^135^ (Figure 5A). The translocation of heme across the IM is carried out by CcmABCD, with CcmE serving as a heme‐trafficking protein to transfer heme to the CcmFGH(I) module, which catalyzes heme ligation to apo‐cyt *c* ^13^, ^136^, ^137^. During translocation, heme is never free.

Unlike CcsBA, CcmABC constitutes an ABC transporter specialized for heme translocation across the IM^132^, ^138^. The structures of CcmABCD solved recently by two teams using Cryo‐EM differed from each other in the composition of the complex, Ccm(AB)_2_CD and Ccm(ABCD)_2_ ^132^, ^139^ (Figure 5A). Despite this, in the transporter, CcmA is a classic cytoplasmic ATPase that binds and hydrolyzes ATP to drive conformational changes in integral membrane proteins CcmB and CcmC^132^. However, consistent with CcsBA, heme translocation by CcmABC does not depend on the energy released from ATP hydrolysis^140^. Although CcmB is the core permease subunit, it is CcmC that has been proposed to carry out heme translocation^132^, ^139^. The notion is largely based on the early findings that CcmC was found to be not only absolutely essential but also sufficient (when in excess) for heme transfer and attachment to CcmE and the fact that CcmC harbors a WWD domain^141^. Nevertheless, direct evidence to support this is missing. More importantly, CcmC lacks a heme channel found in CcsBA^10^, ^142^, implying that the true player for heme translocation in System I remains elusive. Based on the heme ABC transporters that absorb heme from the membrane discussed above, HrtBA and CydDC, the possibility that the immediate heme source of CcmABCD is the membrane could not be ruled out. A bold assumption would be that the heme in the membrane is captured by CcmB and subsequently transferred to the WWD domain of CcmC^132^.

## REGULATION OF HEME SYNTHESIS, ACQUISITION, AND EXPORT BY HEMOPROTEINS

To maintain adequate heme levels and prevent the deleterious effects of excess heme, all cellular processes involved in heme homeostasis must be tightly regulated. In bacteria, regulation predominantly occurs at the transcription level, and a large number of transcriptional regulators have been identified and characterized. A portion of these regulators do not use heme as a cofactor for signal sensing, including iron homeostasis master metalloregulator Fur (ferric uptake regulator), Fnr (fumarate and nitrite reductase regulator), and two‐component system (TCS) ArcBA (aerobic respiratory control), both of which are global regulators in response to intracellular redox levels, LysR‐type transcriptional regulator (LTTR) OxyR (and its functional counterparts), which is the master regulator mediating cellular responses to oxidative stress, extra‐cytoplasmic function (ECF) σ factors, and more^143^, ^144^, ^145^, ^146^, ^147^. As the regulation of heme homeostasis is not the primary role that these regulators play, it is beyond the scope of this review to describe how they work.

**Figure 6:**
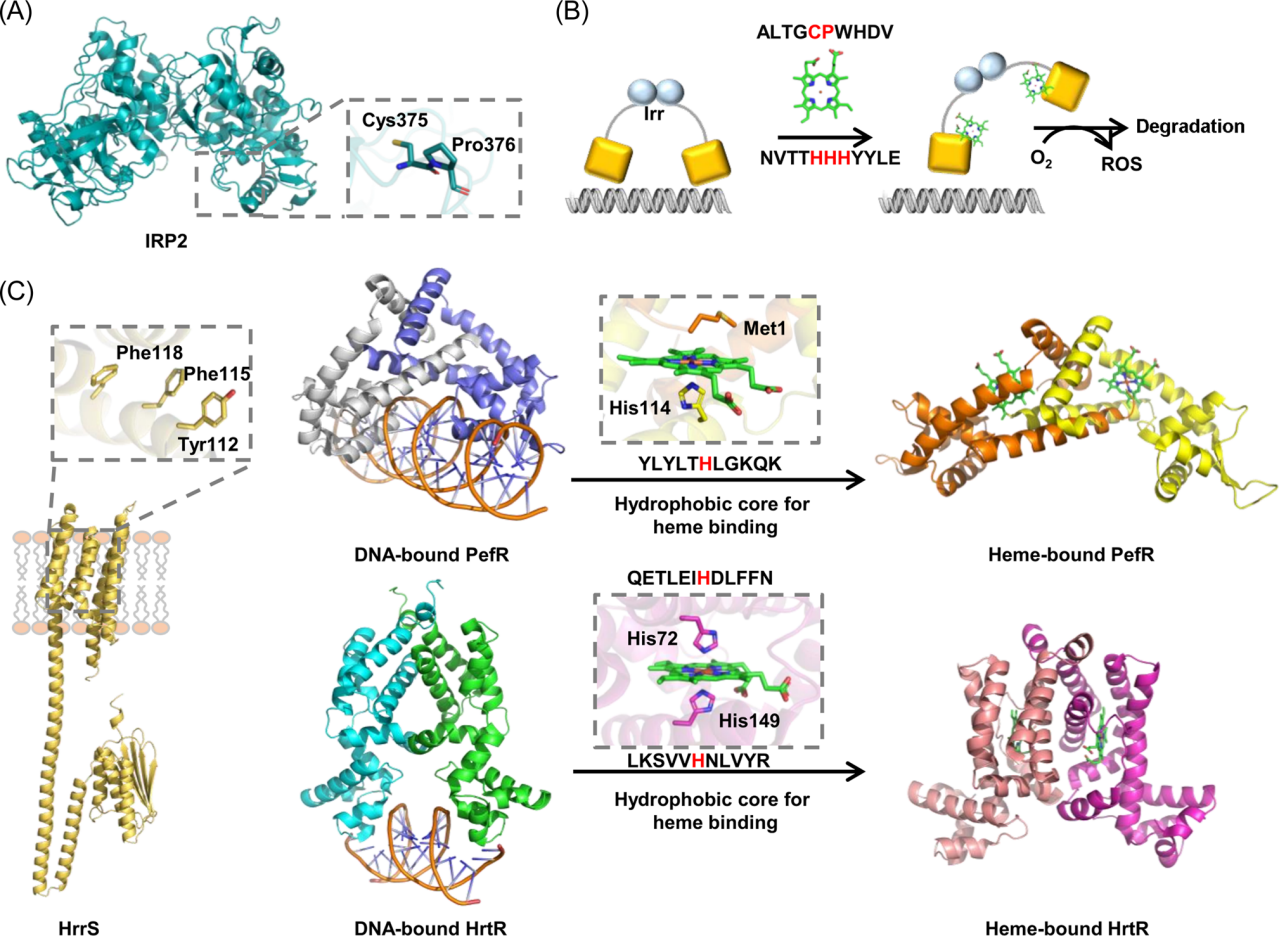
Regulatory hemoproteins containing Cys–Pro (CP) motifs and non‐CP motifs. (A) Representative hemoprotein with a CP motif for heme coordination, IRP2 (PDB ID: 6VCD). (B) Heme‐induced dissociation of Irr from the target gene and oxidative modification. (C) Representative hemoproteins with non‐CP motifs for heme coordination, including PerR, HrtR, and TCS HrrS. Heme‐induced dissociation of PefR and HrtR from the target genes are shown. The structures displayed on the top‐right, top‐left, bottom‐right, and bottom‐left corners, respectively, are *Streptococcus agalactiae* heme‐bound PefR (PDB ID: 7DVR), *S. agalactiae* DNA‐bound PefR (PDB ID: 7DVV), *Lactococcus lactis* heme‐bound HrtR (PDB ID: 3VP5), and *L. lactis* DNA‐bound HrtR (PDB ID: 3VOK). The structure of sensor kinase HrrS is predicted by AlphaFold2^150^. Heme‐binding sites are shown in the close‐up view of the protein structure.

To mediate the expression of the systems for the synthesis, acquisition, and export of heme, the regulators with heme as a cofactor are more specific, even dedicated in many cases. Most of them are from major single‐component transcriptional regulator families and the TCS family. The well‐characterized examples include Irr of the Fur family, PefR of the MarR (multiple antibiotic resistance regulator) family, HrtR of the TetR (tetracycline resistance regulator) family, HbrL of the LTTR family, and HssRS of the TCS family. To respond rapidly to environmental fluctuations, these regulators (or sensor components) bind to the heme transiently with micromolar affinities^117^. The structural characteristics, called “heme‐regulatory motifs (HRMs)”, via which transient interactions take place, are widespread and have been extensively studied^148^, ^149^. The first recognized HRM is called the Cys–Pro (CP) motif, in which the Cys residue functions as an axial ligand to coordinate the heme iron, depending on the Pro residue for stabilization (Figure 6A), and all other motifs identified later are inclusively grouped into the non‐CP family^117^.

Iron response regulator (Irr), initially identified as a Fur‐family transcriptional regulator of heme biosynthesis in *Bradyrhizobium japonicum*, is a global regulator of iron homeostasis and metabolism in nitrogen‐fixing bacteria^151^, ^152^. Irr has two heme‐binding sites: one is a CP motif (Gly28‐**Cys**‐**Pro**‐Trp‐His‐Asp) and the other is the His cluster region (His117–119, non‐CP)^153^, ^154^ (Figure 6B). Under iron‐limiting conditions, Irr represses the transcription of the *hemB* gene, which encodes one of the heme synthetic enzymes, δ‐aminolevulinic acid dehydratase. This repression prevents the accumulation of protoporphyrin IX, a cytotoxic heme precursor to the cell^155^. When the heme concentration is high, the binding of heme to Irr leads to the formation of a stable heme‐bound Irr complex, which can promote oligomerization and down‐regulate DNA‐binding affinity, allowing *hemB* transcription and thus heme biosynthesis^156^. In fact, Irr complexes with the heme biosynthetic enzyme ferrochelatase, and in this way, the regulator is able to respond to the status of the heme at the site of heme biosynthesis^156^. Subsequent investigations have uncovered that the binding of heme triggers the degradation of Irr not bound to DNA by a mechanism that involves a two‐step oxidative modification of the protein^153^, ^157^.

As discussed above, heme efflux systems are exploited as an effective strategy to overcome heme cytotoxicity in Gram‐positive bacteria. To promptly express these systems in response to abruptly increased extracellular heme concentrations, diverse types of transcriptional regulators are harnessed. For instance, PefR and HsmR of the MarR family, as well as HrtR, HatR, and FhtR of the TetR family, function to repress the transcription of heme efflux systems^118^, ^121^, ^122^, ^158^. When in excess, heme binds to these regulators to dissociate them from the target DNA, allowing transcription of the downstream operons, leading to the production of transporters that efflux heme out of the cell^19^. PefR from β‐hemolytic *Streptococcus agalactiae* controls transcription of two operons, *pefAB* and *pefCD*, for a drug:H^+^ antiporter and an ABC multidrug exporter, respectively^121^ (Figure 6C). The pyramid‐shaped homodimer of PefR comprises a hydrophobic heme pocket formed by the α2, α5, and α6 helices from one subunit and the α1 helix from the other subunit, in which the heme is coordinated with His114 and Met1 of the other subunit of the homodimer^19^ (Figure 6C). This unique heme coordination allows a >20 Å structural rearrangement of the DNA‐binding domains such that the dissociation of PefR from the target DNA could take place^19^. Similarly, HsmR from *Clostridium difficile* also forms a homodimer in the canonical conformation shared by MarR family proteins, and His50 has been suggested as the residue coordinating heme^159^. The gene under the direct control of HsmR, *hsmA*, encodes a transmembrane protein that binds to but does not efflux heme. Presumably, the binding results in heme sequestration, which not only detoxifies excess heme but also helps defend against redox stress^159^.

*C. difficile* also encodes TetR‐type HarR, which controls the expression of heme exporter MFS HarT, but mechanical insights into how it interacts with heme remains unavailable^118^. In contrast, within the well‐studied HrtR of *Lactococcus lactis*, which mediates the expression of HrtBA, heme is hexacoordinated by a unique heme‐sensing motif with two histidines: His72 and His149^160^ (Figure 6C). Upon heme binding, HrtR undergoes a coil‐to‐helix transition of the α4 helix in the heme‐sensing domain, inducing a conformational change that dissociates the protein from the target DNA for *hrtBA* de‐repression (Figure 6C). In the case of FhtR (faecalis heme transport regulator) from *Enterococcus faecalis*, which controls the expression of heme‐dependent catalase KatA in addition to HrtBA, at least Thr132 has been found to be critical for heme coordination^122^.

LTTR‐type HbrL from the purple phototrophic bacterium *Rhodobacter capsulatus* also regulates transcription by interacting with the heme. In the absence of heme, enhanced HbrL‐dependent transcription of all *hem* genes is observed^161^, ^162^. It has been demonstrated that HbrL is capable of binding heme, and the binding of the resultant HbrL–heme complex to the target promoters represses transcription. However, further investigations are needed to elucidate the detailed mechanisms of heme binding and heme‐responsive regulation.

In addition to single‐component transcriptional regulators, a few TCSs, including HssRS (heme *s*ensing *s*ystem), ChrSA, and HrrSA, play a critical role in the regulation of some heme‐related processes, heme efflux in particular^117^. HssRS, identified initially in *S. aureus* and conserved in many Gram‐positive bacteria, activates the expression of the heme efflux system HrtBA, which protects bacterial cells from heme toxicity by exporting heme from the membrane, as discussed earlier^119^, ^124^. However, HssS may not sense heme directly, as it lacks a heme‐binding domain. Instead, HssS might sense small toxic molecules that are derived from heme or linked to heme toxicity^163^. The sensing mechanism is further complicated by the crosstalk between HssRS and HitRS, a TCS that is activated by some compounds that impair the integrity of the cell envelope^163^. In contrast, ChrSA and HrrSA, both of which are commonly found in the *Corynebacteriaceae* family either alone or together, sense heme directly in a 1:1 binding stoichiometry per monomer^164^, ^165^. Sensor kinases ChrS and HrrS have a similar configuration, composed of six transmembrane helices, within which a heme‐binding pocket is formed^120^, ^165^, ^166^. Despite this, the binding pockets and coordinating residues are unlikely to be the same: HrrS relied on Tyr112‐Phe115‐Tyr118 for heme binding, whereas ChrS may only use Thr61 for heme coordination^165^ (Figure 6C). In addition, the regulons of these two TCS systems are drastically different. ChrSA acts as a dedicated system for the transcription of the HrtBA heme exporter, while HrrSA appears to be a global regulator of heme homeostasis and beyond^167^, ^168^, ^169^, ^170^. Moreover, given that ChrSA and HrrSA sense the same stimulus and share significant sequence similarity, and presumably a high level of structural similarity, it is conceivable that crosstalk between them could occur^168^, ^169^.

Apart from conventional transcriptional regulators, the HemP/HmuP family represents a unique group of regulators that modulate the expression of the operons for heme uptake and utilization^171^. The members of this family are small heme‐binding proteins, less than 90 amino acid residues in length^171^. In *B. japonicum* and *B. multivorans*, the HemP/HmuP proteins function as transcriptional activators, although they lack a typical DNA‐binding domain^172^, ^173^. This discrepancy may be reconciled by their interaction with Irr and Fur. Nonetheless, there are other possibilities. In *Ensifer meliloti* and *Chromobacterium vioaceum*, these small proteins positively regulate expression at the posttranscriptional level^174^, ^175^.

The final piece of heme homeostasis regulation by hemoproteins in response to heme availability is made up of complex regulatory cascades. A well‐understood example is the HasA–HasR–HasS–HasI system found in *P. aeruginosa* and *S. marcescens*, in which HasA and HasR are hemophore and cognate TBDR, respectively, as mentioned earlier^46^ (Figure 3A). Under normal conditions, HasR holds up anti‐*σ* HasS, which in turn sequesters ECF *σ* HasI, preventing the target genes of HasI from being transcribed^46^. Extracellular heme is sensed by hemophore HasA, which has a high affinity for heme because of the heme pocket surrounded by two coordinating residues, His32 and Thr75, as well as stabilizing residue His83, and then transferred to HasR^46^, ^172^, ^173^, ^174^, ^175^, ^176^. As this heme movement is against the affinity gradient, the axial heme coordination in the hemophore has to be disrupted in the order of His32 and Thr75^46^, ^177^, ^178^. HasR contains a conserved heme receptor motif FRAP (Trp609‐Arg‐Pro‐Pro) and NPNL (Asn634‐Pro‐Phe‐Leu) in *P. aeruginosa* and *S. marcescens*, respectively, to sense the heme, assisted by a histidine residue His624 and His603 in *P. aeruginosa* and *S. marcescens*, respectively^179^. Subsequently, HasR undergoes conformational changes to allow heme binding to its N‐terminal plug domain, including a crucial histidine residue His221 and His189 in *P. aeruginosa* and *S. marcescens*, respectively^180^, ^181^, ^182^. Heme binding then triggers interaction between the plug domain and the anti‐*σ* HasS, resulting in the release of *σ* HasI. In the end, freed HasI binds to the promoters of the target genes, and transcription is allowed^45^.

## CONCLUDING REMARKS

Because of its ubiquity and versality, heme is an essential molecule for virtually all living organisms. Well established as a prosthetic group in enzymes, heme has now been accepted as a signaling molecule that mediates diverse molecular and cellular processes. Here, we summarize the current understanding of heme homeostasis in bacteria, covering biosynthesis, acquisition, utilization/degradation, intracellular trafficking, and export of heme as well as the regulatory systems involved. However, given the enormous breadth of cellular processes linked to heme, neither all the proteins implicated in the subjects nor the mechanisms underlying their physiological roles reported to date can be elaborated upon in the review.

Still, following rapid advances in the heme biology field over the last decade, as presented here, we are more than ever aware of the complexity of heme biology. In fact, in many cases, we are still not certain which substrates, residues crucial for heme binding, and downstream targets are preferred by a given hemoprotein under physiological conditions. Similarly, the extent of heme trafficking protein's function remains to be fully elucidated. It is anticipated that the cellular processes and regulatory roles that involve hemes can still expand, and the questions prompted by new discoveries in the future can be unprecedented. Further efforts will be required to generate knowledge that will enable a better understanding of what governs the biological, biochemical, and structural properties of proteins harnessing heme as a cofactor and beyond.
